# Supplementary material for: Effect of atrial fibrosis on clot burden score and physicochemical properties of thrombus in patients with ischaemic stroke occurring in non-valvular atrial fibrillation
Source: PeerJ. 2025 Mar 24;13:e19173. doi: 10.7717/peerj.19173 (PMC11949112; doi:10.7717/peerj.19173)
Supplement: Supplemental Information 2 [file peerj-13-19173-s002.docx]

group： 1=positive group，CBS(0-6)，

0=negative group，CBS(7-10)

Gender： 2=female

1=male

hypertension、heart failure、vascular disease、age（65-74）、age（＞75）、stroke、diabetes、coronary heart disease ：The data in the table sre CHA2DS2-VASc score

PTFV1＜-0.03 mm·s： 1=positive group

0=negative group

AF type：1=chronic atrial fibrillation

0=paroxysmal atrial fibrillation
